# Aerobic vaginitis, bacterial vaginosis, and vaginal candidiasis among women of reproductive age in Arba Minch, southern Ethiopia

**DOI:** 10.1038/s41598-024-58654-y

**Published:** 2024-04-29

**Authors:** Addis Aklilu, Melat Woldemariam, Aseer Manilal, Getahun Koira, Reham M. Alahmadi, Gurusamy Raman, Akbar Idhayadhulla, Manaye Yihune

**Affiliations:** 1 Department of Medical Laboratory Science, College of Medicine and Health Sciences, Arba Minch University, Arba Minch, Ethiopia; 2 School of Medicine, College of Medicine and Health Sciences, Arba Minch University, Arba Minch, Ethiopia; 3 School of Public Health, College of Medicine and Health Sciences, Arba Minch University, Arba Minch, Ethiopia; 4 Department of Botany and Microbiology, College of Science, King Saud University, P.O. Box 2455, 11451 Riyadh, Saudi Arabia; 5 Department of Life Sciences, Yeungnam University, Gyeongsan, Gyeongbuk-do South Korea; 6 Research Department of Chemistry, Nehru Memorial College (Affiliated to Bharathidasan University), Puthanampatti, Tiruchirappalli District, Tamil Nadu 621007 India; 7 Department of Health Promotion, CAPHRI Care and Public Health Research Institute, Maastricht University, Maastricht, The Netherlands

**Keywords:** Susceptibility, Arba Minch, Bacterial vaginosis, Candidiasis, Lower reproductive tract infections, Immunology, Microbiology, Diseases

## Abstract

Reproductive tract infections (RTIs) are a persistent public health threat worldwide, particularly among women in low-income countries of Africa, including Ethiopia, where drug resistance is also a growing problem. It is crucial to address this problem to ensure women's health and well-being. A cross-sectional study was carried out among a cohort of 398 women of reproductive age who sought medical attention at the Gynecology Department of the Arba Minch General Hospital, southern Ethiopia, from January to June 2020. They were chosen through systematic random sampling, and a pre-tested structured questionnaire was used to collect the data. The collection of vaginal and/or cervical swabs were done to diagnose bacterial vaginosis (BV) and aerobic vaginitis (AV) using Nugent and AV score analyses, respectively. The swabs were subjected to standard microbiological culture techniques to detect the isolates causing AV and vaginal candidiasis (VC). The susceptibility profiles of the causative agents of AV were checked by the Kirby-Bauer disc diffusion technique. Descriptive and inferential statistical analyses were also done. Aerobic vaginitis was the predominantly diagnosed RTI (n = 122, 30.7%), followed by BV (n = 117, 29.4%) and VC (n = 111, 27.9%). The prominent bacteria of AV were *Escherichia coli* (n = 36, 34.2%) and *Klebsiella pneumoniae* (n = 30, 28.5%). The overall rate of multidrug-resistant (MDR) bacteria was 65.71% (n = 69). History of abortion (*p* = 0.01; AOR = 4.0, 95% CI = 2.1, 7.7) and the habit of using vaginal pH-altering contraceptives (*p* = 0.01; AOR = 4.7, 95% CI = 2.5, 8.8) have the greatest odds of RTI. The high prevalence of RTIs in our study warrants an urgent intervention to minimize the associated morbidities and complications. The overall rate of MDR bacterial isolates necessitates the implementation of an effective surveillance program in the study setting.

## Introduction

Infections occurring in the reproductive or genital tracts (RTIs) comprising sexually transmitted diseases, iatrogenic infections, bacterial vaginosis (BV), and vulvovaginal candidiasis (VC) are caused by either exogenous or endogenous agents^1,2^. The World Health Organization (WHO) states that RTIs pose significant public health threats, especially in developing nations^1^. Young women are at greater risk of contracting infections during menstruation, pregnancy, and childbirth^3^. Bacterial vaginosis, aerobic vaginitis (AV), and vaginal candidiasis are indeed the worst hit, with endogenous lower RTIs^4^.

As per a conceptual model developed to study the pathogenesis of BV, *Gardnerella vaginalis* is causing the onset of BV, along with other secondary intruders^4^. Aerobic vaginitis is characterized by an abnormal microflora in the vagina in conjunction with an enhanced local inflammatory reaction and immune response and is often misdiagnosed^5^. The bacterial aetiology of AV is mainly aerobic in nature, or it can be due to facultative anaerobic commensals or pathogens^5^. Recently, the WHO enlisted AV into their guidelines, stressing the importance of accurate diagnosis and prompt treatment^6^. Vaginal candidiasis/moniliasis is the second most common RTIs after BV. The epidemiologic data showed that about 75% of women experienced at least an episode of VC, and 40–50% of them had to face recurrence during later life, which varied significantly across countries, regions, and also among different study populations^7–9^.

Nevertheless, the prevalence of other RTIs in Ethiopia remains the highest in African nations^10^; evidence is scarce concerning the existing rates of lower RTIs, particularly BV, AV, and VC. Unfortunately, routine surveillance of RTIs is not performed in the country, and thus, the estimation of cumulative prevalence is difficult. A literature survey revealed that studies had not yet been conducted in this context in southern Ethiopia, leading to the present attempt.

## Materials and methods

### Study area, period, design, population, and eligibility criteria

This research was conducted in Arba Minch General Hospital from January to June 2020 and is a cross-sectional type, including women of reproductive age, suspected of RTIs, who sought medical care from the Gynecology Outpatient ward. The inclusion criteria were: all women ≥ 18 years who were clinically suspected of RTIs (those who fulfilled at least three of Amsel's criteria for BV; red and edematous vaginal appearance, burning sensation, dyspareunia, thick and mucoid yellow discharge connected to AV and peri-vaginal pruritis, erythema and thick and curdy discharge for VC) and who gave consent to involve in the study. The exclusion criteria were: women who received antibiotics/antifungals during the two weeks before sample collection, those who were extremely unwell and hence could not give answers to questions, and also women who were on a catamenial period. After a thorough briefing of all the study-related procedures and associated risk factors, informed consents were obtained from each participant before their enrolment in the study.

### Sample size determination and patient recruitment

For determining the sample size, a single population proportion formula was used. A prevalence of 48.6% of RTIs was opted from a previous study conducted elsewhere in Ethiopia^11^. By applying a confidence interval of 95% (z = 1.96) and a 5% marginal error (d = 0.05), the sample size calculated was 378. A 10% non-response rate (38 subjects) was applied, and the final sample size thus became 416. The study participants were recruited using a systematic random sampling method.

### Data collection

After the admission of patients to the gynecology ward, they were thoroughly examined by a gynecologist and recruited in conjunction with the clinical criteria of RTIs. Data on socio-demography, medical history, and other pertinent factors were solicited by a face-to-face interview using a pre-tested structured questionnaire. The clinical data corresponding to each participant were collected after reviewing the medical records.

### Sample collection

Samples were meticulously collected from the lateral vaginal wall and posterior fornix using sterile high vaginal cotton swabs moistened with Amie's medium; swabs were placed in Amie's transporting media and quickly taken to the Medical Microbiology and Parasitology Laboratory and stored at 5 °C.

### Microbiological diagnosis of bacterial vaginosis, aerobic vaginitis, and vaginal candidiasis

Vaginal swabs were initially smeared onto clean glass slides and subjected to Gram-staining, and each slide was observed under oil emersion light microscopy with a maximum magnification of 100 × and graded as per the standardized quantitative morphological classification of Nugent score analysis^12^. Briefly mentioning, Gram-stained smears were scored according to a morphotype classification: (a). Lactobacillus morphotypes (score 4 to 0), (b). small Gram-negatives (*G. vaginalis*, score 0 to 4), (c). curved Gram variable rods (Mobiluncus morphotypes, score 0 to 2). The scoring system (0 to 10) is a sum of all the three morphotypes. The diagnostic criterion for bacterial vaginosis was a score of ≥ 7. The diagnosis of AV is made by an ‘AV’ score analysis (a score of ≥ 3 is diagnostic)^6^ and is further substantiated by conventional culture techniques. Cervical swabs were also subjected to Gram-staining to detect the presence of any Gram-positive yeast cells so as to diagnose VC.

### Culture of bacteria (aerobic vaginitis) and yeast-like fungus (vaginal candidiasis)

Swabs were inoculated onto mannitol salt agar, MacConkey, blood agar (5%), and chocolate agar to isolate non-fastidious aerobic bacteria associated with AV. The inoculated plates were incubated aerobically at 37 °C for 24 to 48 h; colonies were identified by Gram-staining and conventional biochemical tests^13^. In the case of suspected VC, cervical swabs were inoculated onto Sabouraud dextrose agar, and isolates were characterized by conventional methods^13^. All the microbiological media used were purchased from HiMedia Laboratories Pvt. Ltd, Mumbai, India.

### Antibiotics susceptibility testing

Antimicrobial susceptibility testing of non-fastidious aerobic bacteria was performed by the Kirby-Bauer disc diffusion technique on Mueller Hinton agar (Oxoid, Basingstoke, Hampshire, UK), according to the guidelines set by the Clinical Laboratory Standard Institute (CLSI)^14^. Multidrug-resistant (MDR) bacteria in this study were extrapolated as those resistant to ≥ 3 classes of antibiotics tested^15^.

### Data quality assurance

The structured questionnaire was pre-tested on 5% of the sample size in Chencha General Hospital. The data collectors and technicians were given sufficient training sessions as per standard procedures. The data were checked daily for accuracy, clarity, and completeness, and any incompleteness and errors found were immediately corrected with utmost confidence. Standard operating procedures (in-house SOP manual) were followed during the collection, transportation, and processing to maintain the highest level of quality. All reagents, culture media, and antibiotic discs were carefully inspected for their shelf life and physical condition and were stored at 2–8 °C. The culture media were incubated at 37 °C overnight to ensure sterility until actual sample processing. The efficacy of the media was determined by inoculating the American Type Culture Collection (ATCC) (*S. aureus* (ATCC 25923) *E. coli* (ATCC 25922), *P. aeruginosa* (ATCC 27853), and *Candida albicans* (ATCC 10231)).

### Statistical analyses

The collected data were coded, cleaned, and entered using Epi-Data version 4.2 and then exported to SPSS version 25 software for further analysis. The IBM SPSS Statistics for Windows, version 25 (IBM Corp., Armonk, N.Y., USA) was used for the data analysis, and descriptive statistics, including frequency, mean, and standard deviations, were done. To evaluate the association among variables and RTIs, bivariable and multivariable logistic regression analyses were applied. In the former model, variables with a *p* value < 0.25 were selected, whereas in the latter case, a *p* value ≤ 0.05 was considered statistically significant.

### Ethical considerations

Ethical clearance was approved by the Institutional Review Board, College of Medicine and Health Science, Arba Minch University (Ref. IRB/150/12, Dated 19-12-2019). This study was in line with the declaration of Helsinki and its later amendments. Before the enrolment, each participant completed and signed a consent form indicating their willingness.

## Results

### Socio-demographic characteristics

The study included a cohort of 398 participants, with a response rate of 95.67% (out of 416). Most of them were within the age group of 25 to 30 (n = 165, 41.5%), and more than half of them were married (n = 207, 52%). Many of them had a history of previous sexual intercourse, and of these, the majority had only one heterosexual partner (n = 227, 57%) (Table 1).

**Table 1:** Socio-demographic, clinical and obstetrics characteristics of women of reproductive age.

Variables		Prevalence n (%)		Bivariate analysis	
Variables	Categories	Positive	Negative	*P* value	COR (95% CI)
Residence	Urban	180 (50.6)	176 (49.4)	1	
	Rural	18 (42.9)	24 (57.1)	0.34	1.36 (0.71–2.6)
Age	18–24	36 (35.6)	65 (64.4)	0.20	1.50 (0.79–2.83)
	25–30	93 (56.4)	72 (43.6)	0.13	0.64 (0.36–1.15)
	31–35	39 (59.1)	27 (40.9)	0.12	0.57 (0.29–1.15)
	≥ 36	30 (45.5)	36 (54.5)	1	
Marital status	Married	93 (44.9)	114 (55.1)	1	
	Single	57 (65.5)	30 (34.5)	0.62	0.87 (0.52–1.47)
	Divorced	9 (39.1)	14 (60.9)	0.001	0.42 (0.25–0.72)
	Widowed	39 (48.1)	42 (51.9)	0.59	1.26 (0.52–3.06)
Educational level	Illiterate	24 (40.0)	36 (60.0)	0.21	1.5 (0.79–2.84)
	Read and write only	33 (61.1)	21 (38.9)	0.18	0.64 (0.32–1.24)
	Primary school	42 (44.2)	53 (55.8)	0.41	1.26 (0.72–2.19)
	Secondary school	45 (55.6)	36 (44.4)	0.45	0.8 (0.44–1.42)
	College and above	54 (50.0)	54 (50.0)	1	
Occupational status	Student	12 (25.5)	35 (74.5)	1	
	Employee	63 (50.0)	63 (50.0)	0.00	0.34 (0.16–0.72)
	Merchant	12 (33.3)	24 (66.7)	0.43	0.68 (0.26–1.78)
	Homemaker	57 (59.4)	39 (40.6)	0.00	0.23 (0.10–0.50)
	Labour worker	39 (54.2)	33 (45.8)	0.00	0.29 (0.13–0.64)
	Others*	15 (71.4)	6 (28.6)	0.00	0.13 (0.04–0.43)
Number of sexual partners	Only one	108 (47.6)	119 (52.4)	1	
	Two	45 (55.6)	36 (44.4)	0.18	0.71 (0.42–1.18)
	Three	18 (40.0)	27 (60.0)	0.38	1.33 (0.69–2.5)
	Four and above	24 (66.7)	12 (33.3)	0.03	0.44 (0.21–0.92)
Monthly income of the family (ETB)	< 1000	36 (40.4)	53 (59.6)	0.31	0.63 (0.26–1.53)
	1000–2000	102 (55.7)	81 (44.3)	0.01	0.34 (0.15–0.78)
	2000–5000	52 (53.6)	45 (46.4)	0.03	0.38 (0.16–0.91)
	> 5000	9 (30.0)	21 (70.0)	1	
Frequency of changing the panties	Once per day	135 (46.6)	155 (53.4)	1	
	1 panty for 2–3 days	51 (58.6)	36 (41.4)	0.49	0.61 (0.37–0.99)
	1 panty for 4 or above days	12 (57.1)	9 (42.9)	0.35	0.65 (0.26–1.5)
Frequency of douching	Once a day	42 (48.3)	45 (51.7)	1	
	Twice a day	93 (56.7)	71 (43.3)	0.20	0.7 (0.42–1.2)
	Three times per day	21 (30.4)	48 (69.6)	0.03	1.98 (1.0–3.8)
	4 or more per day	36 (57.1)	27 (42.9)	0.31	0.71 (0.36–1.3)
	Others*	6 (40)	9 (60)	0.55	1.4 (0.45–4.2)
History of RTI/STI	Yes	126 (54.5)	105 (45.5)	0.02	0.63 (0.42–0.94)
	No	72 (43.1)	95 (56.9)	1	
Type of chronic Disease	HIV	51 (41.5)	72 (58.5)	0.04	2.75 (1.02–7.42)
	Diabetes	39 (72.2)	15 (27.8)	0.02	3.53 (1.28–9.71)
	Hypertension	6 (40)	9 (60)	0.96	0.96 (0.31–2.94)
	Multiple Chronic diseases	6 (40)	9 (60)	0.06	3.75 (0.92–15.22)
	Others**	15 (71.4)	6 (28.6)	1	
Parity	0	60 (51.7)	56 (48.3)	1	
	1–3	108 (51.4)	102 (48.6)	0.95	1.01 (0.64–1.5)
	4 and more	30 (41.7)	42 (58.3)	0.18	1.5 (0.82–2.7)
Pregnancy	Yes	138 (52.7)	124 (47.3)	0.10	0.71 (0.47–1.08)
	No	60 (44.1)	76 (55.9)	1	
Previous hospitalization (six months)	Yes	72 (49.3)	74 (50.7)	0.89	1.03 (0.68–1.54)
	No	126 (50)	126 (50)	1	
History of abortion	Yes	93 (56.7)	71 (43.3)	0.02	0.62 (0.42–0.93)
	No	105 (44.9)	129 (55.1)	1	
Smoking habit	Yes	54 (60)	36 (40)	0.03	0.59 (0.36–0.94)
	No	144 (46.8)	164 (53.2)	1	
History of catheterization	Yes	30 (47.6)	33 (52.4)	0.71	1.12 (0.65–1.89)
	No	168 (50.1)	167 (49.9)	1	
Previous antibiotics usage^a^	Yes	96 (55.2)	78 (44.8)	0.05	0.68 (0.46–1.01)
	No	102 (45.5)	122 (54.5)	1	
Corticosteroid drug usage	Yes	36 (60)	24 (40)	0.08	0.61 (0.35–1.07)
	No	162 (47.9)	176 (52.1)	1	
Usage of vaginal pH-altering contraceptives	Yes	129 (54.4)	108 (45.6)	0.02	0.63 (0.42–0.94)
	No	69 (42.9)	92 (57.1)	1	
History of rape	Yes	48 (51.6)	45 (48.4)	0.68	0.91 (0.57–1.44)
	No	150 (49.2)	155 (50.8)	1	

*Farmer, Commercial sex worker.

**Don't wash per day; wash after urination.

***Hepatitis and Tuberculosis, *ETB* Ethiopian Birr.

^a^Antibiotics usage in the past six months, *STI* sexually transmitted infection, *CD* chronic diseases, *HIV* Human immunodeficiency virus, *RTI* Reproductive tract infection, *COR* Crude odds ratio, *CI* Confidence interval, n: number of positive or negative cases, *%* percent, *1* reference group.

### Obstetrics, clinical, and hygienic characteristics

The majority have the habit of changing their panties daily (n = 290, 72.9%), douching (rinsing) twice a day, and had a history of RTIs/STIs as well as chronic diseases, i.e., 58% (n = 231) and 57.3% (n = 228), respectively. Also, a considerable number of women with chronic diseases had HIV infection (n = 123, 30.9%); most of them (n = 237, 59.5%) used some kind of pH-altering vaginal contraceptives.

### Prevalence of reproductive tract infections

**Figure 1:**
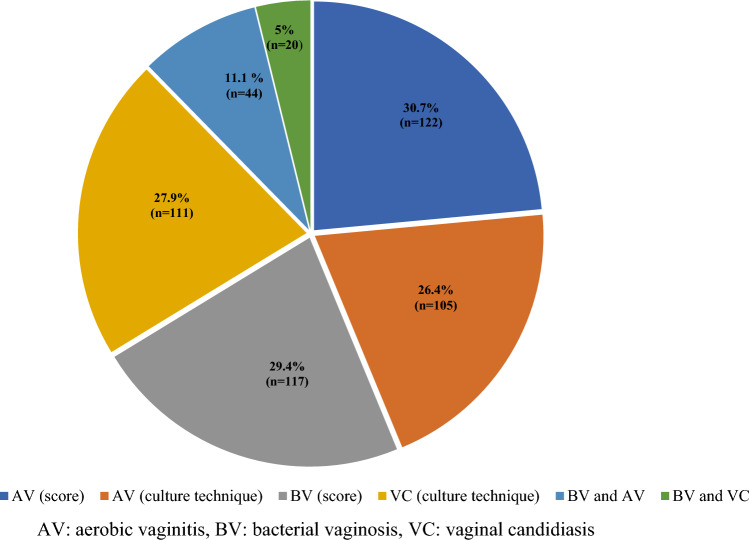
Profile and percentage of reproductive tract infections.

The overall prevalence of RTIs was 49.7% (n = 198) (95% CI 44.7, 54.8). The extent of BV and AV diagnosed by Nugent and AV scoring criteria was 29.4% (n = 117) (95% CI 25, 34.1) and 30.7% (n = 122) (95% CI 26.2, 35.4), respectively. However, compared to the AV score, the number of positive cases detected by the culture technique was slightly lower, i.e., 26.4% (n = 105) only. On the other hand, the prevalence of VC was 27.9% (n = 111) (95% CI 23.5, 32.6) (Fig. 1). The rate of co-infections was 11.1% (n = 44) (95% CI 8.1, 14.6) for BV/ AV and 5% (n = 5) (95% CI 3.1, 7.7) for BV /VC.

### Bacterial profile of aerobic vaginitis

**Figure 2:**
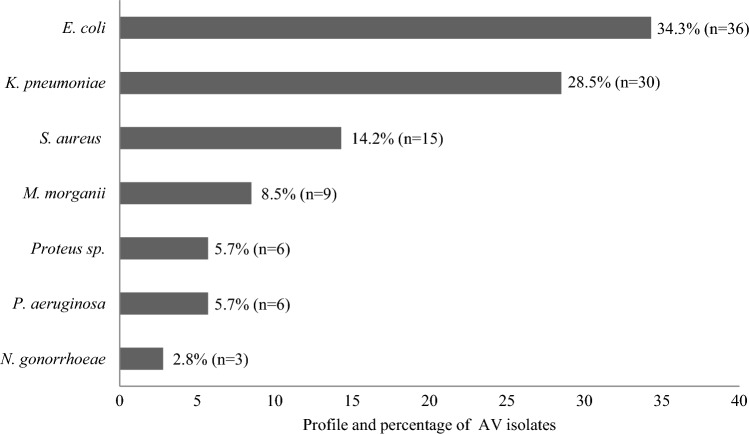
Profile and percentage of bacterial isolates associated with aerobic vaginitis (AV).

In total, 105 (n) isolates were detected; most of them were Gram-negative (n = 90, 85.7%), whereas Gram-positives accounted for only a total of 14.2% (n = 15). The predominant Gram-negative isolate was *E. coli* (n = 36, 34.3%), followed by *K. pneumoniae* (n = 30, 28.5%). The isolates of *S. aureus* were the only Gram-positive bacteria (n = 15, 14.2%) detected in this study (Fig. 2). Of notice, 2.8% of women were diseased with gonococcal vaginitis, and is an alarming fact.

### Antimicrobial susceptibility profiles of aerobic vaginitis

The overall susceptibility profiles of bacteria against sixteen antibiotics were summarized in Table 2. More than half of the isolates of *E. coli* were resistant against amoxicillin-clavulanic acid (66.6%) and sulfamethoxazole-trimethoprim (58.3%). The isolates of *K. pneumoniae* also showed a higher extent of resistance against meropenem (70%) and ceftriaxone (60%), whereas 90% of them were susceptible to ciprofloxacin. The isolates of *P. aeruginosa* were exclusively susceptible to ciprofloxacin, gentamicin, amikacin, piperacillin, and norfloxacin. On the other hand, 50% of these isolates were resistant to a pair of antibiotics, namely meropenem and cefepime. All the isolates of *S. aureus* were resistant to cefoxitin (100%) and sulfamethoxazole-trimethoprim (100%). Invariably, all *S. aureus* isolates were 100% susceptible to gentamicin and clindamycin (Table 2); out of the entire isolates obtained 69 (65.71%) were MDR. The MDR patterns correspond to 100% of *Proteus* sp., 100% of *S. aureus*, and 75% of *E. coli* (Table 3).

**Table 2:** Antimicrobial susceptibility patterns of bacterial isolates.

Antibiotic	Patterens	Bacterial isolates of AV n (%)							
		*E. coli* (n = 36)	*K. pneumoniae* (n = 30)	*Proteus* spp. (n = 6)	*M. morganii* (n = 9)	*P. aeruginosa* (n = 6)	*N. gonorrhoeae* (n = 3)	*S. aureus* (n = 15)	Total
P	S	NT	NT	NT	NT	NT	–	–	–
	I						3 (100)	–	3 (100)
	R						–	15 (100)	15 (100)
AUG	S	6 (16.7)	3 (10)	–	6 (66.7)	NT	NT	NT	15 (18.5)
	I	6 (16.7)	15 (50)	–	3 (33.3)				24 (29.6)
	R	24 (66.6)	12 (40)	6 (100)	–				38 (51.9)
PIP	S	NT	NT	NT	NT	6 (100)	NT	NT	6 (100)
	R					–			–
SXT	S	15 (41.7)	15 (50)	6 (100)	9 (100)	NT	NT	–	45 (46.8)
	R	21 (58.3)	15 (50)	–	–			15 (100)	51 (53.2)
CXT	S	NT	NT	NT	NT	NT	NT	–	–
	R							15 (100)	15 (100)
CTX	S	27 (75.0)	15 (50)	6 (100)	9 (100)	NT	–	NT	57 (67.9)
	R	9 (25.0)	15 (50)	–	–		3 (100)		27 (32.1)
CTR	S	3 (8.3)	9 (30)	–	3 (33.3)	NT	NT	NT	15 (18.8)
	I	18 (50)	3 (10)	–	3 (33.3)				24 (30.0)
	R	15 (41.7)	18 (60)	6 (100)	3 (33.3)				42 (52.2)
CFP	S	NT	NT	NT	NT	–	NT	NT	–
	I					3 (50)			3 (50)
	R					3 (50)			3 (50)
MER	S	6 (16.7)	–	–	3 (33.3)	3 (50)	NT	NT	12 (13.8)
	I	15 (41.7)	9 (30)	–	3 (33.3)	–			27 (30.0)
	R	15 (41.7)	21 (70)	6 (100)	3 (33.3)	3 (50)			48 (55.2)
GEN	S	15 (41.7)	12 (40)	3 (50)	9 (100)	6 (100)	NT	15 (100)	60 (58.8)
	I	6 (16.7)	6 (20)	–	–	–		–	12 (11.8)
	R	15 (41.7)	12 (40)	3 (50)	–	–		–	30 (29.4)
AMK	S	22 (61.1)	15 (50)	3 (50)	9 (100)	6 (100)	NT	NT	55 (65.6)
	I	14 (38.8)	12 (40)	3 (50)	–	–			29 (31.0)
	R	–	3(10)	–	–	–			3 (3.4)
CHL	S	30 (83.3)	18 (60)	6 (100)	9 (100)	NT	NT	15 (100)	78 (81.2)
	R	6 (16.7)	12 (40)	–	–			–	18 (18.8)
CIP	S	30 (83.3)	27 (90)	6 (100)	9 (100)	6 (100)	–	12 (80)	80 (85.7)
	I	–	–	–	–	–	3 (100)	–	3 (2.9)
	R	6 (16.7)	3 (10)	–	–	–	–	3 (20)	12 (11.4)
NOR	S	NT	NT	NT	NT	6 (100)	NT	NT	6 (100)
	R					–			–
ERY	S	NT	NT	NT	NT	NT	NT	6 (40)	6 (40)
	R							9 (60)	9 (60)
CLI	S	NT	NT	NT	NT	NT	NT	15 (100)	15 (100)
	R							–	–

*AUG* amoxicillin-clavulanic acid (20/10 µg), *AMK* amikacin (10 μg), *CFP* cefepime (30 μg), *CTX* cefotaxime (30 μg), *CTR* ceftriaxone (30 μg), *CXT* cefoxitin (30 µg), *MER* Meropenem (10 μg ), *CLI* clindamycin (30 µg), *ERY* erythromycin (15 µg), *GEN* gentamicin (10 μg), *CIP* ciprofloxacin (5 μg), *NOR* norfloxacin (30 µg), *P* penicillin (1 unit), *SXT* sulfamethoxazole-trimethoprim (1.25/23.75 µg), *PIP* piperacillin (100 µg), *CHL* chloramphenicol (30 μg), *NT* not tested, *R* resistant, *I* Intermediate, *S* susceptible.

*NT corresponds to a change in the denominator (total number of isolates tested), *AV* Aerobic vaginitis.

**Table 3:** Multidrug resistance profiles of bacterial isolates.

Bacterial isolates	R3	R4	R5	R6 and above	MDR
	n (%)				
*E. coli* (n = 36)	12 (44.5)	–	9 (33.3)	6 (22.2)	27 (75)
*K. pneumoniae* (n = 30)	6 (28.5)	–	3 (14.2)	12 (57.1)	21 (70)
*Proteus* spp. (n = 6)	3 (50)	3 (50)	–	–	6 (100)
*S. aureus* (n = 15)	–	6 (40)	9 (60)	–	15 (100)
Total	21 (29.2)	9 (12.5)	21 (30.8)	18 (25)	69 (65.7)

*R3* resistant to 3 antibiotics, *R4* Resistant to 4 antibiotics, *R5* resistant to 5 antibiotics, *R6* resistant to 6 antibiotics, *MDR* Multidrug resistance, *n* number, *%* percentage.

### Associated factors of reproductive tract infections

During the bivariable logistic regression analysis, variables such as age, educational level, marital status, occupation, number of sexual partners, family income, history of RTI, chronic diseases, parity, pregnancy, history of abortion, smoking habit, history of antibiotics, consumption of corticosteroids and also the usage of vaginal pH-altering contraceptives were statistically significant with respect to RTIs. However, in multivariable logistic regression analyses, only two variables, namely the history of abortion (*p* = 0.01; AOR = 4.09, 95% CI = 2.16, 7.73) and the usage of vaginal pH-altering contraceptives (*p* = 0.01; AOR = 4.71, 95% CI = 2.51, 8.83) showed statistical association with RTIs (Table 4).

**Table 4:** Multivariable analysis of RTI with socio-demographic, clinical and obstetrics characteristics.

Variables	Categories	Prevalence n (%)		Multivariable analysis	
		Positive	Negative	*P* value	AOR (95% CI)
Residence	Urban	180 (50.6)	176 (49.4)		
	Rural	18 (42.9)	24 (57.1)	–	–
Age	18–24	36 (35.6)	65 (64.4)	0.07	2.15 (0.93–4.98)
	25–30	93 (56.4)	72 (43.6)	0.55	0.82 (0.41–1.62)
	31–35	39 (59.1)	27 (40.9)	0.12	0.52 (0.23–1.19)
	≥ 36	30 (45.5)	36 (54.5)	1	
Marital status	Married	93 (44.9)	114 (55.1)	1	
	Divorced	57 (65.5)	30 (34.5)	0.01*	1.55 (0.38–6.40)
	Widowed	9 (39.1)	14 (60.9)	0.01*	1.45 (0.33–6.41)
	Single	39 (48.1)	42 (51.9)	0.24	0.43 (0.11–1.76)
Educational level	Illiterate	24 (40.0)	36 (60.0)	0.83	1.16 (0.32–4.26)
	Read and write only	33 (61.1)	21 (38.9)	0.41	0.59 (0.16–2.11)
	Primary school	42 (44.2)	53 (55.8)	0.69	1.23 (0.43–3.49)
	Secondary school	45 (55.6)	36 (44.4)	0.52	1.39 (0.51–3.76)
	College and above	54 (50.0)	54 (50.0)	1	
Occupational status	Student	12 (25.5)	35 (74.5)	1	
	Employee	63 (50.0)	63 (50.0)	0.00*	3.43 (0.56–20.82)
	Merchant	12 (33.3)	24 (66.7)	0.01*	7.09 (1.48–33.95)
	Homemaker	57 (59.4)	39 (40.6)	0.04*	5.44 (1.03–28.51)
	Labour worker	39 (54.2)	33 (45.8)	0.95	1.04 (0.27–4.02)
	Others^a^	15 (71.4)	6 (28.6)	0.39	1.87 (0.45–7.86)
Number of lifetime sexual partners	One	111 (47)	119 (53)	1	
	Two	45 (55.6)	36 (44.4)	0.59	0.58 (0.08–4.24)
	Three	18 (40.0)	27 (60.0)	0.03*	2.25 (0.58–7.67)
	Four and above	24 (66.7)	12 (33.3)	0.01*	1.75 (0.46–6.71)
Monthly income of the family (Ethiopian birr)	< 1000	36 (40.4)	53 (59.6)	0.95	0.96 (0.25–3.69)
	1000–2000	102 (55.7)	81 (44.3)	0.26	0.51 (0.15–1.67)
	2000–5000	52 (53.6)	45 (46.4)	0.23	0.49 (0.15–1.58)
	> 5000	9 (30.0)	21 (70.0)	1	
Frequency of changing the panties	Once per day	135 (46.6)	155 (53.4)		
	1 panty for 2–3 days	51 (58.6)	36 (41.4)	–	–
	1 panty for 4 and above days	12 (57.1)	9 (42.9)	–	–
Frequency of douching	Once a day	42 (48.3)	45 (51.7)		
	Twice a day	93 (56.7)	71 (43.3)	–	–
	Three times per day	21 (30.4)	48 (69.6)	–	–
	4 or more per day	36 (57.1)	27 (42.9)	–	–
	Others^b^	6 (40)	9 (60)	–	–
History of RTI/STI	Yes	126 (54.5)	105 (45.5)	0.46	0.75 (0.35–1.59)
	No	72 (43.1)	95 (56.9)	1	
Chronic diseases	HIV	51 (41.5)	72 (58.5)	0.13	3.14 (0.72–13.69)
	Diabetes mellitus	39 (72.2)	15 (27.8)	0.02*	4.01 (0.93–17.26)
	Hypertension	6 (40)	9 (60)	0.94	0.95 (0.19–4.67)
	Multiple CD	6 (40)	9 (60)	0.02*	1.13 (0.13–10.02)
	Others^c^	15 (71.4)	6 (28.6)	1	
Parity	0	60 (51.7)	56 (48.3)	1	
	1–3	108 (51.4)	102 (48.6)	0.00	0.09 (0.03–0.27)
	4 and more	30 (41.7)	42 (58.3)	0.01	0.32 (0.14–0.73)
Pregnancy	Yes	138 (52.7)	124 (47.3)	–	–
	No	60 (44.1)	76 (55.9)		
History of hospitalization	Yes	72 (49.3)	74 (50.7)	–	–
	No	126 (50)	126 (50)		
History of abortion	Yes	93 (56.7)	71 (43.3)	0.01*	4.09 (2.16–7.73)
	No	105 (44.9)	129 (55.1)	1	
Smoking habit	Yes	54 (60)	36 (40)	0.19	0.54 (0.21–1.38)
	No	144 (46.8)	164 (53.2)	1	
History of catheterization	Yes	30 (47.6)	33 (52.4)	-	-
	No	168 (50.1)	167 (49.9)		
History of antibiotics usage	Yes	96 (55.2)	78 (44.8)	0.03	0.49 (0.28–0.94)
	No	102 (45.5)	122 (54.5)	1	
Use of corticosteroid	Yes	36 (60)	24 (40)	0.33	0.65 (0.28–1.55)
	No	162 (47.9)	176 (52.1)	1	
Usage of pH-altering contraceptives	Yes	129 (54.4)	108 (45.6)	0.01*	4.71 (2.51–8.83)
	No	69 (42.9)	92 (57.1)	1	
History of rape	Yes	48 (51.6)	45 (48.4)	–	–
	No	150 (49.2)	155 (50.8)		

^a^Farmer, Commercial sex worker, ^b^Don't wash per day; wash after urination; ^c^Hepatitis, TB, *(statistically significant at *p* < 0.05), *AOR* Adjusted odds ratio, *CI* confidence interval, *n* number of positive or negative cases, *%* percent, *1* reference group, *f* frequency, – (not eligible), *STI* sexually transmitted infection, *CD* chronic diseases, *HIV* Human immunodeficiency virus, *RTI* Reproductive tract infection.

## Discussions

This is the first study done among women of reproductive age suspected of RTIs in Arba Minch, which underscores the importance of research and its impact on their health. The overall prevalence of RTIs was 49.7%, highlighting the necessity of serious management of the issue. The types of RTIs and the aetiological agents found by us are different from those reported in recent literature. In other words, each study is unique, and the scenario and results obtained need not be an exact replica of previous studies. For instance, recent studies on RTIs carried out in other parts of the country showed a wider prevalence range, 15.6—50%, corresponding to varied aetiology^16,17^. The higher prevalence found in our study warrants an urgent intervention so that associated morbidities and complications can be minimized. It is well recognized that RTIs are a persisting problem, particularly in low-income countries of Africa. A failure in prompt diagnosis and treatment can lead to various pregnancy-related complications as well as congenital infections. Recently, it has been shown that RTIs can also increase the chances of HIV transmission^18^. Ensuring routine screening for and treatment of RTIs in women is crucial for the prevention of complications and for maintaining economic productivity and quality of life. Focusing on the high-risk group must be done but by giving equal importance to regular screening for all women of reproductive age. In general, the prevalence of AV is 7–12%, and it is less than that of BV^19^. However, in our study, as per the scoring criteria, AV was the most frequently diagnosed RTIs (30.7%). The positivity rate was higher than the results reported in an earlier study done in another part of the country (22.9%)^16^, as well as in Belgium (7.9%)^12^. Vulvovaginal candidiasis is one of the trivial reasons for medical, nursing, and pharmacist consultations, and this substantiates our findings that vaginal candidiasis was the second most common RTI affecting 27.9%; this result tally with earlier findings from Nepal (25%)^20^; VC is often linked to the recent use of antibiotics, progesterone-containing oral contraceptives, or immunosuppression^21^. The exact cause behind the excessive growth of endogenous microbes is still being investigated; prompt diagnosis and treatment can avoid the risk of recurrence. A recently published article thoroughly reviewed the epidemiology and risk factors linked to BV^22^. We found that BV was the third most frequently diagnosed RTI, with a prevalence as high as 26.3%, which is even higher than that reported earlier from Bahir Dar (2.8%)^17^. On the other hand, the prevalence of BV now found in Arba Minch is substantially below the extent reported in other cities of the country, Gondar and Addis Ababa (35.5–48.6%)^11,16^, and also Gabon (62.8%)^23^. The prevalence of BV can fluctuate widely due to varying diagnostic criteria, alterations in the characteristics of clinical populations enrolled, and some mismatching factors in the study populations. It is envisaged that severe infections are usually poly-microbial in nature, whereas milder infections are generally mono-microbial. In our study, 11% of cases correspond to AV and BV co-infection.

The pathogenic microbiota in the vagina of AV patients is very complex, and precise detection of aetiological agents is challenging but vital for the better management of the cases. The Gram-negative isolates were the prominent group of causative agents of AV, as in the case of previous studies from Gondar^17^ and Addis Ababa^11^. The dominant one of this kind found in our study was *E. coli,* 34.2% (n = 36), which is at par with the outcome of an earlier study done in the capital city^11^. *Klebsiella pneumoniae* was the second most frequently isolated bacteria, as is the case in an earlier study from Addis Ababa^11^. The frequently isolated Gram-positive bacteria in the current study was *S. aureus,* which supports the notion that many species of bacteria can colonize both the gastrointestinal and reproductive tract and can become the source or reservoir^24^; however, their clinical relevance is yet to be elucidated.

We found that 85% of Gram-negative bacteria were susceptible to ciprofloxacin, whereas 81.2% were susceptible to chloramphenicol. This is in agreement with a couple of studies from Bahir Dar^17,25^; the latter drug is generally not recommended for the treatment of RTIs. The overall resistance rates of Gram-negative bacteria against sulfamethoxazole-trimethoprim, ceftriaxone, and amoxicillin-clavulanic acid were above 50% and are likely to shoot up further if ignored, resonating with the outcome of a previous study from the country^17^. Among the aerobic Gram-negative bacteria*, E. coli* was the most resistant organism; the resistance was very high against amoxicillin-clavulanic acid (66.6%) and sulfamethoxazole-trimethoprim (58.3%) and is in line with the outcome of a couple of studies conducted in the country^11,17^. Also, the susceptibility profiles of *K. pneumoniae* to ciprofloxacin and chloramphenicol are similar to the results of earlier work conducted in Ethiopia itself^16^.

A dubious finding of our study is related to the higher resistance of *K. pneumoniae* against meropenem compared to ceftriaxone, although the former drug is not in regular use in our study setting in the case of RTIs; perhaps the unscrupulous usage of this drug in other patients in our hospital would have resulted in this increased resistance. This ambiguity should be cleared and verified by secondary investigations. The variations in the resistance profiles of *K. pneumoniae* against ceftriaxone in comparison with meropenem appear odd, and to arrive at a convincing conclusion, several future studies are to be conducted continuously, including a larger number of study participants across the country; also, it needs further in-depth studies involving molecular techniques. This phenomenon was also detected in the case of *Klebsiella oxytoca* (carbapenem-resistant but ceftriaxone and cefepime-sensitive) in a study done in the USA^26^.

Isolates of *S. aureus* showed the highest extent of resistance (100%) against penicillin and sulfamethoxazole-trimethoprim and matched well with the results of an earlier study done in Addis Ababa^11^. Alarmingly, all these isolates were found to be methicillin-resistant too, which is quite contrary to a few studies done in the nation earlier^11,16^. Although Ethiopia is registered with the Global Antimicrobial Resistance Surveillance System, nationwide studies are sorely lacking. The most alarming observation in this study is that 65.71% of the isolates were MDR, which is much higher than the values reported in a couple of cities in the country^16,27^. A higher level of multidrug resistance was also observed, particularly in the case of isolates of *S. aureus*, *Proteus* sp. (each 100%), *E. coli* (75%), and *K. pneumoniae* (70%); this matches with the findings described in a systematic review which concluded that these isolates are highly resistant to the most of the frequently used antibiotics^27^. Endogenous vaginal infections are often misdiagnosed as STIs, such as trichomoniasis, gonorrhea, and chlamydia leading to the unwanted prescription of antibiotics. The high rate of MDR observed in our study setting could be due to the excessive reliance on empirical therapy, which can be modified by taking into account the current sets of results.

In our setting, ciprofloxacin and ceftriaxone are the first and second-line drugs of treatments for AV, respectively. The antibiogram profile revealed that 11.4 and 52.2% of the bacteria were resistant to these antibiotics, respectively, making the latter drug less effective for an empirical therapy. These results are quite relevant and can be considered to tailor the local antibiotic policy from time to time. Our results also bring a notion that women who are colonized with MDR pathogens are at greater risk of contracting nosocomial infections, highlighting the need for further extensive studies.

Abortion and the usage of vaginal contraceptives were only statistically significant, as noted in some of the preceding studies^10,28^. We observed that women with a history of abortion are four times more prone to develop RTIs, and this can be attributed to the fact that operative procedures render women more susceptible to infections. Similarly, women who were using vaginal contraceptives were four times more prone to develop RTIs because of fluctuation of pH, which increases the risk of developing VC or BV. To mitigate this issue, modern contraceptive methods, including oral, injectable (medroxyprogesterone), skin implant (Nexplanon), and skin patch (progestin and estrogen), may be promoted.

While precisely enlisting the impact of this research, we stress that enlightening women about the seriousness of RTIs and encouraging them to seek immediate medical assistance is very important. Additionally, there must be augmented vigilance from the side of clinicians in distinctly diagnosing the cases of AV and BV and prescribing the exact regimen. Also, they should instruct the patients to maintain a high level of alert post-abortions as well as while using pH-altering vaginal contraceptives.

### Limitations

The molecular characterization of bacterial and fungal isolates could not be performed due to the inadequacy of advanced techniques; pathogenicity and virulence factors of bacteria also were not studied. In addition, the identification of fastidious organisms and antifungal susceptibility testing of *C. albicans* was not performed due to the lack of infrastructure/ facilities. We have employed only conventional techniques for the diagnosis of RTIs; therefore, alternate diagnostic tools are required to figure out the reasons for the negative results obtained. Additionally, the study population was hospital-based, and hence, the results may be skewed to a certain extent. A comprehensive multicentric study is required to confirm some of the current findings. We are unable to establish a clinical correlation between the aetiology of AV with the isolated pathogens. Another limitation is the lack of resistant gene analysis of *K. pneumoniae* against ceftriaxone and meropenem. Finally, we add that data on differential diagnosis are not included.

## Conclusions

This is the first report on the prevalence of RTIs among women of reproductive age attending the Arba Minch General Hospital in Ethiopia. The AV, VC, and BV were found to be the common RTIs among them. Isolates of *E. coli*, *K. pneumoniae,* and *S. aureus* were the predominant bacteria causing AV; an alarming finding is that 65.71% of bacterial isolates causing AV were MDR. It is better to initiate an antimicrobial stewardship program aiming at optimizing the selection of drugs in our study setting to prevent untoward consequences. The history of abortions and the usage of pH-altering vaginal contraceptives were statistically associated. Women in the reproductive age group must be enlightened to reduce the burden of infections. The stakeholders and policymakers should give due attention to the management of RTIs by reinforcing the prevention and control strategies through an early promotion of screening and the initiation of treatment if required.

## Data Availability

The datasets used and/or analysed during the current study available from the corresponding author on reasonable request.
